# Fairness of Financial Contribution in Iranian Health System: Trend Analysis of National Household Income and Expenditure, 2003-2010

**DOI:** 10.5539/gjhs.v7n5p260

**Published:** 2015-03-16

**Authors:** Amir Abbas Fazaeli, Hesam Seyedin, Abbas Vosoogh Moghaddam, Alireza Delavari, H. Salimzadeh, Hasan Varmazyar, Ali Akbar Fazaeli

**Affiliations:** 1 Departments of Health Economics, School of Health Management and Information Sciences, Iran University of Medical Sciences, Tehran, IR Iran; 2 Iranian Social Security Organization, Tehran, IR Iran; 3 Health Policy Making Secretariat, Ministry of Health and Medical Education/NCD Research Center, Endocrine and Metabolism Research Institute, Tehran University of Medical Sciences and Health Services, Tehran, Iran; 4 Digestive Diseases Research Center, Shariati Hospital, Tehran University of Medical Sciences and Health Services, Tehran, Iran; 5 Health Management and Economics Research Center, Iran University of Medical Sciences, Tehran, Iran

**Keywords:** Fairness in Financial Contribution, Catastrophic Health Expenditure, Iran, health services, equity

## Abstract

**Background:**

Social systems are dealing with the challenge of achieving fairness in the distribution of financial burden and protecting the risk of financial loss. The purpose of this paper is to present a trend analysis for the indicators related to fairness in healthcare’s financial burden in rural and urban population of Iran during the eight years period of 2003 to 2010.

**Methods:**

We used the information gathered by statistical center of Iran through sampling processes for the household income and expenditures. The indicators of fairness in financial contribution of healthcare were calculated based on the WHO recommended methodology. The indices trend analysis of eight-year period for the rural, urban areas and the country level were computed.

**Results:**

This study shows that in Iran the fairness of financial contribution index during the eight-year period has been decreased from 0.841 in 2003 to above 0.827 in 2010 and The percentage of people with catastrophic health expenditures has been increased from 2.3% to above 3.1%. The ratio of total treatment costs to the household overall capacity to pay has been increased from 0.055 to 0.068 and from 0.072 to 0.0818 in urban and rural areas respectively.

**Conclusion:**

There is a decline in fairness of financial contribution index during the study period. While, a trend stability of the proportion of households who suffered catastrophic health expenditures was found.

## 1. Introduction

The importance and impact of healthcare services on the length and quality of life is significant. Also, health and health systems affect economic production. For example, financing system of health through insurance or employment-based insurance may hinder labor mobility and macro-economic performance. In addition, there is increasing evidence that improvements in health can enhance economic growth (Murray & Frenk, 2000).

The economic issues related to healthcare have been studied for several decades, but the fairness in contribution to heath expenditures made by different sections of the population, is a new issue that has been notified by the experts in economy and social sciences in the recent decade. The World Health Organization (WHO), in 2000 report, communicated the idea that the health system is a social subsystem and should follow three main goals: improving the level and equity in distribution of the population health measures to the acceptable standard; increasing the level of responsiveness to the non-medical expectations of the population and making sure that this measures is fairly distributed and enforcing an equitable financial contribution by the population for having access to the healthcare(Braveman, Starfield, & Geiger, 2001).

Definition of the goal, “fairness in financial contribution”, strengthens an equitable cost sharing in health system by all the people whether they are healthy or unhealthy and poor or rich. In other word, everyone irrespective of their economic status or income level, should access to the standard health services in order to live with a highest attainable level of mental and physical health (Moghaddam et al., 2013). Achieving fairness in the distribution of the financing burden and protecting the risk of money loss is a common challenge of social systems around the world (Braveman et al., 2001). It is also the main government responsibility and a prerequisite for sustainable development in each society.

Fortunately, articles 3, 29 and 43 of the Islamic Republic of Iran’s constitutional law has recognized the health and access to health services as a basic needs and a universal human right that should be supported for every individual (Moghaddam et al., 2013).

Ministry of Health and Medical Education of Iran (MOHME) started several strategic studies such as burden of diseases, national health accounts, utilization of health services, assessing fairness in financial contribution and responsiveness to non medical expectations of clients by technical supports of the WHO-EMRO at 2001 (Moghaddam, et al., 2013). These activities were aimed to prepare a realistic situational analysis and evidence based solutions to be recommended in the next 4^th^ 5 years social, economical and cultural development plan (4^th^ 5YDP, 2005 to 2010) that should target the Iran vision 2025. The vision is going to lead the country to a developed state with the highest rank of economic, scientific and technological status in the region. Enjoying health, welfare, food security, social security, equal opportunities, fair income distribution, strong family structure; removing poverty, corruption, and discrimination; and benefitting desirable living environment are characteristics of Iranian society in that year.

Based on the invaluable information and recommendations of this pioneer study, Iran developed appropriate national policies and strategies including: the article 90 in 4th 5YDP and poverty reduction strategy (Mehrara & Fazaeli, 2010). The article 90 says that: “In order to increase equitable access to health services and reducing the shares of vulnerable and low income families in health expenditures, distributional policies of the health resources and facilities should be modified by the state in a way to achieve the following strategic goals: 1) increase the FFCI to 0.90; reducing share of the population from total health expenditure to maximum 30% and reducing the percentage of household facing CATHE to 1% (Economic)

Policies and interventions to attain the above mentioned goals were:

1) Statistical Center of Iran (SCI) should report the analysis of the health financing indices such as FFCI, CATHE, OOP, Health Expenditures based on International standards of National Health Account at national and provincial level, every year, before preparation of annual budget.
2) Health services franchise should be set by the state concurrent to the determination of insurance premium and tariffs annually.
3) Diagnostic and medical protocols, especially for high cost and common ones should be prepared and approved by the medical services insurance high council, for at least 20 items annually. Medical insurance organizations and companies should reimburse the health service providers based on these protocols.
4) In order to cover the catastrophic health expenditures (CATHE) of the vulnerable families by the state, the estimated funds should be put in the annual budget.
5) All the public, university, army, social security organization, private and charity hospitals and diagnostic and medical centers have to provide the drugs, medical devices and consumables for treating hospital inpatients and deduce only the inpatient franchise from insured patients(Razavi, 2011)

For the first time, Razavi et al. estimated the indices of fairness in financial contribution of health expenditures in Iran, based on the WHO protocol, for the years 1995 to 2002. The results showed that the Fair financial Contribution Index (FFCI) and percentage of population facing to CATHE had changed respectively from 0.834 to 0.833 and 2.13% to 2.32% (Xu & Organization, 2005)

In order to produce the comparable trends of the leading indicators of fairness in financial contribution in health expenditures, this article tries to illustrate how much the goal oriented approved interventions in the health system have met the prerequisites of supreme documents such as 4th 5YDP.

## 2. Material and Methods

The WHO protocol for calculating the indices trend uses in order the results is comparable to the national and especially international studies (Xu et al., 2007).

### 2.1 Database

The national database of Statistical Centre of Iran (SCI) on urban and rural household’s income and expenditure survey which is conducted annually was used. In the survey a questionnaire with four sections including demographic, kind of residency and the equipments, consumable and non-consumable costs, and household income is used. The costs related to health issues include four subgroups: drug and medical equipments; out patient’s services; in-patients services and detoxification and réhabilitation.

### 2.2 Sampling

The annual survey under the supervision of SCI is performed in all the provinces in both urban and rural areas using two stage cluster sampling method. The sampling units are Iranian households. The sampling frame consists of blocks (a group of houses which has been limited to street in each side) on the first stage and the houses as the second stage of sampling. The blocks are selected randomly and in each block five houses are approached based on the systematic clock-wise cyclic method. The sample size for the whole study period (2003 to 2010) at national level varies from 23134 to 38170 for each year.

All the data are gathered based on a unique/standard method of face to face interview. The interviewers are well-educated and skillful and the procedures are checked systematically by the field supervisors. The comprehensive descriptive of the survey method has been reported elsewhere (Murray et al., 2003).

### 2.3 Indices Calculation

In this research, the values of the indicators, FFCI and CATHE, were computed for the 8 years period, 2003-2010. In order to produce the indices, we extracted the following data from the whole national household’s income and expenditure survey database:

- Household size: the number of individuals living together as household in a house.
- Gross household and Consumable costs: the total costs which the household paid for the foods and accommodations (except alcoholic drinks, cigarettes, accommodation in hotels and restaurants).
- Out-of-pocket cash paid for health care services (OOP): the total costs that the household paid for the health care services excluded the costs for transportation and the costs which the insurance companies support.
- Capacity to pay (CTP): the total costs of the household subtracted from subsistence expenditures. In cases which the subsistence expenditure exceed the total costs, the CTP was calculated by subtracting the total costs of the household from the food costs.
- Household Financial Contribution proportion (OOPCTP): defined as the household’s total cash paid directly for the health care services (OOP) dividing by the capacity to pay (CTP).
- OOPCTP_h_= OOP_h_/CTP_h_ (h: the household identification code)
- W_h_: Household weighting variable

The Fair Financial Contribution Index (FFCI) was calculated by the following **formula:**


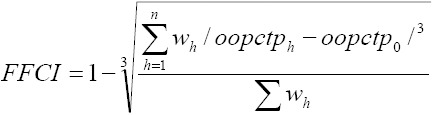


FFCI is an indicator for the financial equity. It was constructed to vary from 0 to 1; the fairer the health financing system, the closer FFC will be to 1.

In the context of OOPCTPh, as this indicator is increased, the household are forced to reduce other living costs in order to save the money for buying health care services. In such condition, many families decrease their payments to recreational activities which subsequently reduce their welfare and quality of life. There is a critical cutoff for this reduction. The catastrophic health expenditure (CATHE) has been set as 40% or more of the household capacity to pay.

### 2.3 Analysis

All the data were calculated and analyzed by STATA software version 10. The OOP, CTP, OOPCTP, FFCI and CATHE indices computed for each urban and rural household. Then, summery of such indices was calculated by year and nationally to explore the temporal and special distribution of the most popular equity indices in Iran.

## 3. Results

**Figure 1:**
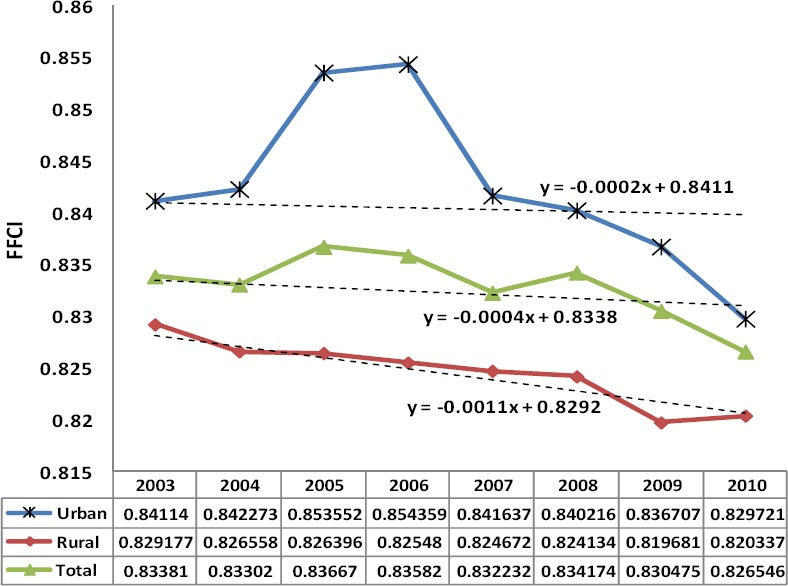
The average of FFCI by living area and year in Iran

Figure 1 shows the trends of FFCI at urban, rural and national levels from 2003 to 2010. In this period, the value of FFCI for the urban and rural areas respectively has been decreased from 0.8411 to 0.8297 and 0.8291 to 0.8203.

**Figure 2:**
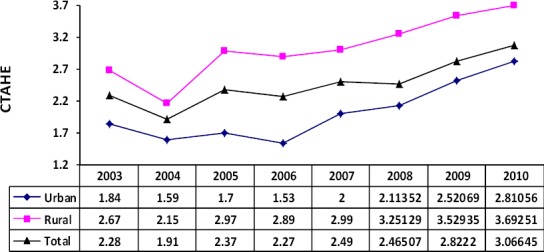
Proportion of the population facing catastrophic health expenditure (CATHE) by living area and year in Iran

CATHE for the eight-year period is shown in Figure 2. The proportion of people who encountered catastrophic health related costs at the national level gradually has been increased from 2.3% (2003) to 3.1% (2010).

## 4. Discussion

As the Figure 1 show, the FFCI has improved in urban areas during 2003 to 2006, but the total trends and the difference between rural and urban, has been decreased specially from 2006. These findings are consistent with Razavi et al results (Razavi, 2011) (14). National figures of FFCI changed from 0.834(2003) to 0.827(2010) in comparison of 0.835(1995) to 0.833(2002). It means that the FFCI reduction has been 3.5 times worse, in the same 8 years period.

The trend was illustrated by line chart and it was analyzed by trend analysis functions and tested by linear regression model. As the Figure 1 shows, the FFCI in rural areas has decreased faster than urban areas from 2007 to 2010. In other words, the FFCI (dependent value) has decreased in each year with 0.02% units in urban areas and 0.11% units in rural areas.

It is noteworthy that in the cities the share of some expenses such as housing in comparison to the total household non-food costs is more than rural areas. Therefore, the relative growth in the items such as housing expenses can cause the reduction in the ratio for health related cost. This can be applied to the non-food expenditure in general. The high rate of inflation in Iran, particularly for the non-food goods will lead to increase in total non- food expenditure compared to the total expenses for the household and causes the decrease in the ratio of health related costs.

The FFC index is sensitive to the average share of health related costs in the total households pay capacity, change in horizontal distribution of share of health related costs in the total households pay capacity (horizontal equity) and change in vertical distribution of share of health related costs in the total households pay capacity (vertical equity). However one cannot determine whether the improvement of this index is because of improvement in health expenditure distribution between deferent income groups (vertical equity) or it is the result of improvement in health expenditure distribution between deferent household in same group (horizontal equity). Also it is not clear whether the average of health cost compared to the total household costs has increased or it has decreased. To remove these ambiguities, other intermediary indicators are computed. These indicators are: the proportion of people who encounter catastrophic health related costs and the proportion of treatment costs to the total household pay capacity (Xu et al., 2003)

According the result of this study the percentage of people with catastrophic costs of health in the urban areas has been reduced from 1.8% (2003) to 1.5% (2006) and then the trend has inversed and reached to 2.8% (2010). Whereas in rural areas this index had a decreasing trend until 2004, in 2005 increased a little and the increasing trend was restarted from 2007 again. In aggregate, the proportion of people who encounter CATHE in rural areas during 2003 to 2010 has increased from 2.66% to 3.7% unfortunately.

On the other hand, consistent with Razavi et al.’ results (11) incremental trend of national figures of CATE from2.1% (1995) to 2.3% (2002) has continued from 2.37% (2005) to 3.07% (2010).

## 5. Conclusion

Our analysis showed that the goal oriented interventions in the Iran’s healthcare system has not led to an improving trend in fairness of financial burden of the health care cost on different sections of the population. Therefore, the recent policies and interventions in health system have not met the goals of article 90 of the five year plan for the 4th 5YDP. Moreover, existence of significant gaps between the indicators for the rural areas and the urban areas show the need for review, prioritization, and corrections of these purposeful interventions based on specific priorities. One of the main focuses of the policy interventions is proposed to be in the rural areas of the country which significantly improve the level of fairness in financial contribution to the health care.
